# Characteristics of Impulse Carburization LPC Process

**DOI:** 10.3390/ma14154269

**Published:** 2021-07-30

**Authors:** Ryszard Filip, Kamil Ochał, Kamil Gancarczyk, Wojciech Jerzy Nowak, Barbara Kościelniak, Bartek Wierzba

**Affiliations:** Department of Materials Science, Faculty of Mechanical Engineering and Aeronautics, Rzeszow University of Technology, Powstancow Warszawy 12, 35-959 Rzeszow, Poland; kochal@prz.edu.pl (K.O.); KamilGancarczyk@prz.edu.pl (K.G.); wjnowak@prz.edu.pl (W.J.N.); b.koscielnia@prz.edu.pl (B.K.); bwierzba@prz.edu.pl (B.W.)

**Keywords:** low-pressure carburizing, Pyrowear53, hardness, residual stresses, carbon concentration

## Abstract

In the present work, Pyrowear53 steel was subjected to the impulse carburizing LPC process. After carburation, the material was quenched and tempered. Postprocessing analyses included the measurement of hardness, carbon content, residual austenite, and residual stresses. The results revealed that the thermochemical treatment resulted in the formation of an approximately 1200 µm wide carburized layer. The results of hardness, carbon content, and residual austenite measurement showed a continuous gradient (drop) in the measured values within the carburized layer. However, the results of residual stresses revealed the existence of a local extremum, namely, a zone with higher compressive stresses at the depth between 600 and 1000 µm. This was explained by a different temperature for initiation of martensite transformation as a function of carbon content. This difference resulted in the occurrence of two martensite expansion fronts at two different depths, resulting in an increase in compressive stresses at the noted depth range. Moreover, it was concluded that this region was present for material containing between 0.8 and 0.4 wt% carbon for Pyrowear53.

## 1. Introduction

Carburizing and hardening processes are the most important processes in the materials economy. Almost all of the existing devices include gears or other hardened components. These gears are treated first by the carburizing process and then hardened in oil, soles, nitrogen gas, or other media. The carburizing temperature varies from 870 to 940 °C; the gas atmosphere for carburizing is produced from liquid or gaseous hydrocarbons such as propane, butane, or methane [1,2]. The study of process parameters in metals during heat treatment has been of considerable interest for some years [3,4,5,6], but there has been relatively little work on process variables during the surface hardening process [7] since controlling parameters in carburizing is a complex problem. The major influencing parameters in carburizing are holding time, carburizing temperature, carbon potential, and quench time in oil [8].

Heavy-duty gearboxes used in aviation are usually made of high-strength alloy steels such as AMS 6308D. In this paper, the influence of the cyclic carburizing process in low pressure carburizing of Pyrowear53 steel on the diffusion process is discussed.

The chemical composition of Pyrowear53 is presented in Table 1 [9,10]. The measured contents of the elements in Pyrowear53 are in accordance with the nominal composition of AMS 6308D.

In the past, Pyrowear53 steel was studied in terms of carburization. A few attempts at modeling the low-pressure carburization process have also been conducted. It was shown that good concurrence between modeled and measured values of distortion and residual stress can be achieved using simulation with the commercial finite-element-based package—DANTE [11,12]. However, most of the literature manuscripts deal with the determination of the carburization recipe to find the proper carbon layer. The basic idea is to develop the proper simulation model. These models are based on diffusion phenomena and proper determination of the diffusion flux of carbon during the process. Such models allow for determination the number of carbon and diffusion cycles during the process and allow for controlling the number of carbides generated during carburization. The model describing carburization of multicomponent alloys was proposed by Bongartz et al.; their work was based on the second Fick law with a composition-dependent diffusion coefficient [13,14,15]. Modeling of the carburization process, when the local equilibrium condition holds, implies the compact carbon layer formation. Therefore, models involving this factor are based on the diffusion equation and the Wagner model [16,17,18,19,20,21].

The aim of this work is to present the influence of carbon cyclic diffusion and the subsequent oil hardening process on the microstructure and residual stress depth profiles. Thus, the experimental results of carbon concentration, hardness, residual austenite, and stress as a function of distance are presented. This process was previously modeled using the bivelocity method. The detailed description can be found elsewhere [22].

## 2. Materials and Methods

In this paper, the rectangular cube shape samples with dimensions 20 × 20 × 10 mm^3^ were prepared from the Pyrowear53 steel. The samples were inserted into a vacuum furnace in order to carburize. They were carburized, quenched, and tempered. The temperature for carburizing was 925 °C. After carburizing, the samples were austenitized at the temperature of 910 °C and quenched in oil. The subzero treatment was performed after quenching. The tempering was performed at the temperature of 200 °C in nitrogen gas atmosphere. Saturation and diffusion times for carburizing were computed using numerical simulation of the mass diffusion mathematic model based on the Darken approximation [3]. The vacuum carburizing followed by quenching and tempering was carried out to achieve a 0.9–1.2 mm effective case depth. The carburized case depth was measured with the use of an Innovatest Nexus 4000 microhardness tester (INNOVATEST, Maastricht, The Netherlands) with Vickers indenter HV 0.5 (INNOVATEST), and GD-OES depth profiling (Horiba Jobin Yvon, Paris, France). Elastic deformation and retained austenite were determined using a Proto iXRD COMBO portable diffractometer (Proto Mfg. Ltd., Oldcastle, ON, Canada). For the measurement of residual stresses as a function of depth, the electrolytic polishing method was used with a step of 100 µm using a Proto Manufacturing Electriolithic 8818-V3 polisher (Proto Mfg. Ltd.). To calculate internal stresses, the sin2ψ method [23] was applied with the assistance of Win2.0 XRD software. The sin2ψ method is based on the Bragg–Brentano symmetrical diffraction and the appropriate elastic constants, and uses a Ψ–type goniometer, which allows for obtaining appropriate inclinations of the diffraction vector by Ψ angles in a plane perpendicular to the diffraction plane [24]. A lamp with a chrome anode of characteristic Cr Kα radiation of 0.2291 nm wavelength was used due to the radiation source of 20 kV, 4 mA with a beam diameter of 2 mm. The effective depth of X-ray beam penetration was 5 µm. The measurement was taken on the surface of the specimen. After the measurement, the surface was electropolished for the removal of 100 µm of the material and the measurement repeated. By this procedure, a plot of measured residual stresses as a function of depth with the step of 100 µm was produced. Residual stresses were determined for constant values of Ψ angle in the range from 25° to −25°. The diffraction line {211} of martensite phase was analyzed for residual stresses in the subsurface region. In the measurement of residual stresses, the values of X-ray elastic constant used for calculations were ^1^/_2_S_2_ = 5.8 × 10**^−^**^6^ MPa and −S_1_ = 1.34 × 10**^−^**^6^ MPa. The quantity of retained austenite was measured for diffraction lines {200}, {211} of martensite and {220}, {200} of austenite phases. After heat treatment and standard procedure for preparation of metallographic cross-sections, the samples were examined using a Hitachi S3400 N scanning electron microscope (Hitachi, Tokyo, Japan) coupled with an energy dispersive spectrometer (EDS). Images were captured in the back-scattered electron (BSE) mode, which enabled the increase of the contrast between the phases. The chemical composition of the phases present in the carburized material was measured using EDS. The dimensions of the phases identified were determined using the NIS-Elements software based on SEM/BSE images. The depth profiles of the samples after the carburizing process were performed using a Horiba JobinYvon glow discharge optical emission spectrometer (GD-OES). The GD-OES depth profiles were quantified using the procedure described in [25,26,27].

## 3. Results

**Figure 1:**
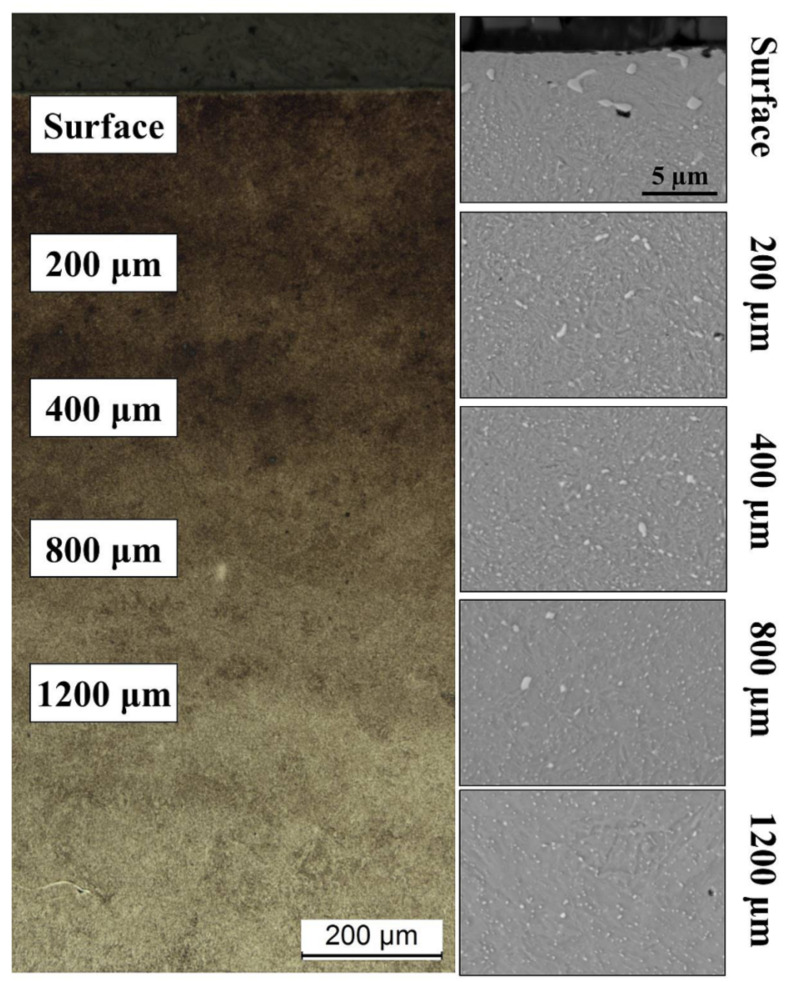
Microstructure of carburized Pyrower53 observed using an optical microscope (**left**) and magnifications observed at given depth using a scanning electron microscope (**right**).

The microstructure of the cross-section of a carburized sample is presented in Figure 1. The microscopic examination showed the presence of massive and globular carbides at the surface of the steel substrate and the presence of fine carbides inside the carburized layer, which was high-carbon martensite. The formation of massive carbide precipitates indicates that the steel was supersaturated with carbon in the subsurface area (Surface). The presence of fine carbides inside and at the boundaries of austenite grains was also observed (depth of 200 and 400 µm). The microstructure in the sample core was tempered martensite with uniformly dispersed alloy carbides containing mainly molybdenum (depth of 800 µm). Residual austenite was present in amounts that cannot be estimated with a light or scanning microscope.

**Figure 2:**
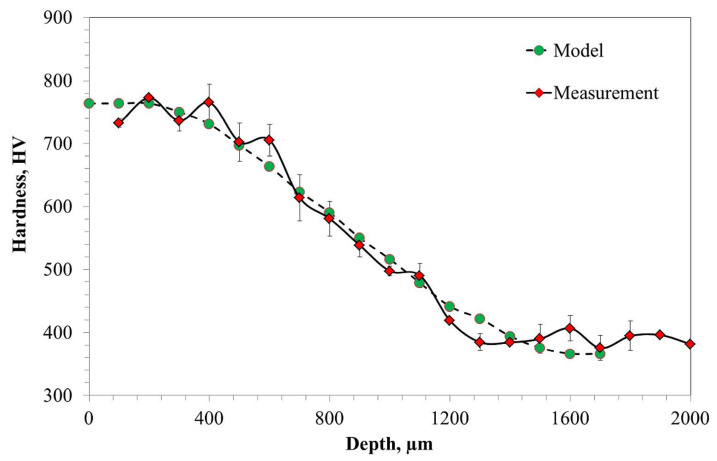
Profile of measured and modeled hardness of Pyrower53 steel after cyclic carburizing process.

After the low pressure carburizing process, hardness, carbon concentration profile, residual austenite, and stresses as a function of distance were measured. The hardness profile is typical for carburized steel. The profile starts with the plateau and hardness decreases with the distance from the surface of the sample. The highest hardness value was obtained in the subsurface region (up to 300 µm, Figure 2) of the sample. The maximum value of hardness in this region was approximately 780 HV, whereas the lowest was measured at the core of the sample, approximately 1300 µm, and the value was about 350 HV. The hardness profile of Pyrower53 steel after the cyclic carburizing process is presented in Figure 2. A similar hardness profile on carburized Pyrowear53 was obtained by A. Wojtyczka and B. Iżowski [20]. Moreover, the measured values of hardness are in very good agreement with those modeled using Equation (1).

**Figure 3:**
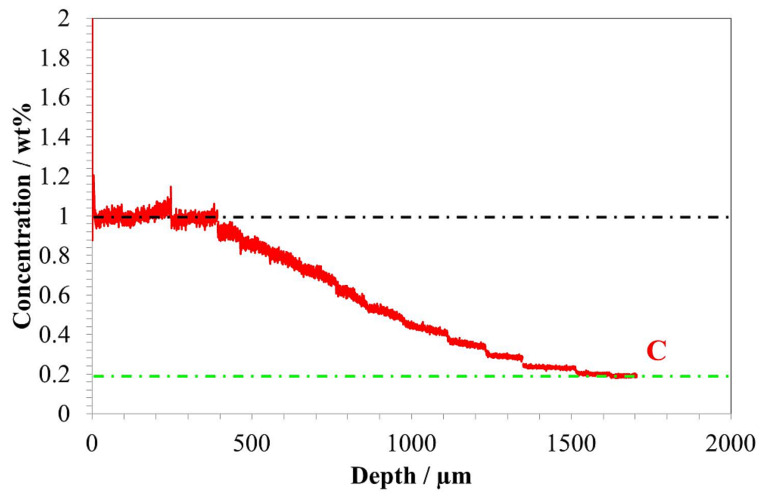
GD-OES concentration profile as a function of distance from the surface.

Along with hardness measurement, the GD-OES concentration profile as a function of depth was performed. As demonstrated in the GD-OES depth profile shown in Figure 3, the highest carbon concentration was measured in the subsurface region of the sample up to approximately 400 µm, where the carbon concentration is 1 wt%; the carbon concentration then decreased with depth. The lowest concentration was observed at the depth of 1500 µm and equal to 0.2 wt%. This value is very close to the initial concentration of carbon in the Pyrower53 steel, Figure 3.

**Figure 4:**
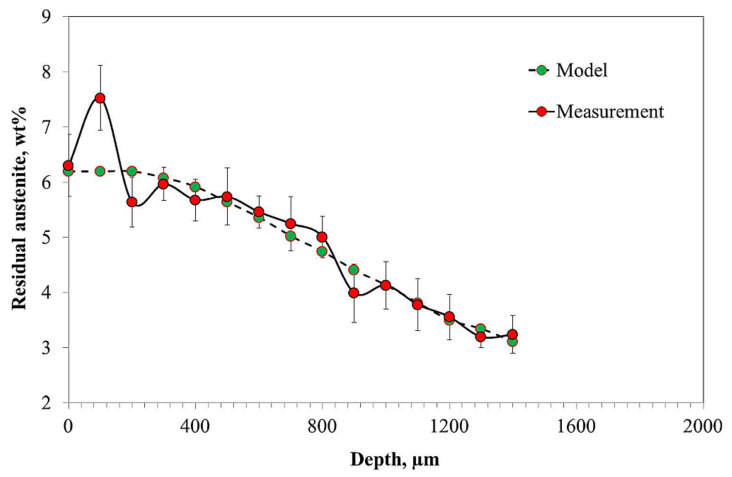
Measured and modeled residual austenite content profile as a function of distance from the surface expressed in weight percent.

During the hardening process, austenite transforms into martensite. However, during this phase transformation some residual austenite remains in the alloy’s microstructure. This residual austenite content should decrease with decrease of carbon content. The experimental and modeled (using Equation (2)) results of residual austenite content are shown in Figure 4. At the surface, the residual austenite content is the highest and decreases with depth and carbon concentration. The highest value at the surface is 7.5 wt%, and at the core residual austenite is 3.2 wt%. As can be observed, the measured and modeled values are in good agreement.

**Figure 5:**
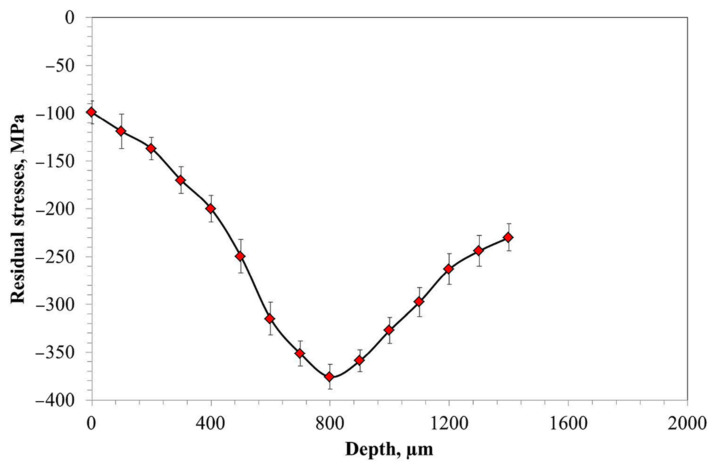
Residual stress profile as a function of distance from the surface.

Figure 5 shows the residual stress as a function of depth. Residual stresses were measured in the center of the sample in two directions (σ_x_ and σ_y_). The values of residual stresses at each depth were averaged. It is clearly shown that the lowest compressive stresses are present in the subsurface region of the sample. The measured value of residual stress at this location is about −100 MPa. As shown in Figure 5, an increase in depth results in a decrease of the value of measured stress (i.e., increase in compressive stress) to approximately −375 MPa at 800 µm. Beyond that depth, the function ascends.

**Figure 6:**
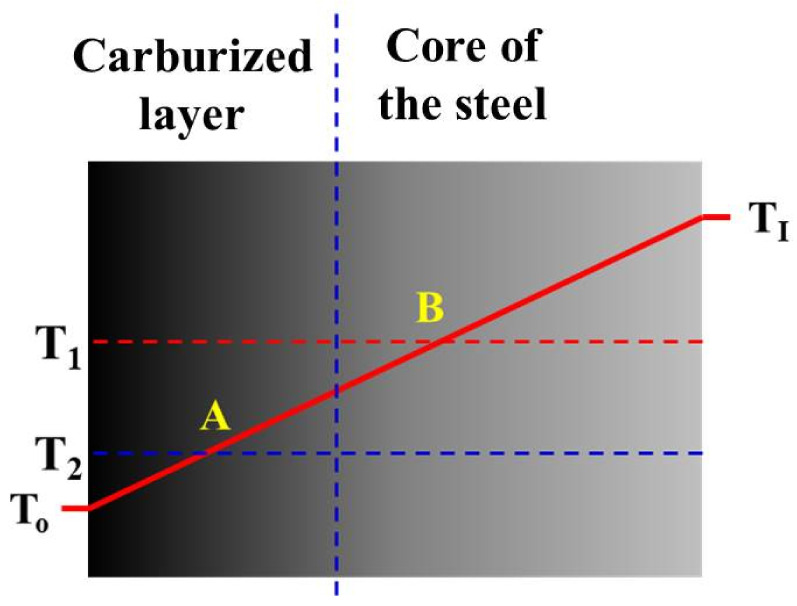
Schematic representation of temperature gradient in the subsurface region of carburized material. T1 and T2 specify the start of the austenite–martensitic transformation temperature; carbon content is shown by points A and B. The gray-shade gradient indicates variation of carbon content in the steel.

The main objective of these studies was to observe the effects on the surface and carbon layer and to determine the relationship with other process parameters while also contributing to the larger picture of carburizing and quenching. The correlation between the composition profile of carbon, hardness profile, stress measurement, and microstructure was shown in this study. After case hardening, compressive residual stresses were found as expected. The global minimum was observed under the stress distribution. This situation arises when the two fronts of austenite–martensitic transformation occurred, Figure 6. It was found that there is dependence between the hardness and concentration profiles. This dependence for Pyrower53 steel can be expressed by linear function:

H = 468C_C_ + 296 (1)

where H is the hardness in HV 0.5 and C_C_ is the carbon concentration in wt%.

R squared fit parameter calculated for Equation (1) was equal to 0.98. This function allows for determining the hardness profile from the concentration profile. A similar approximation can be proposed for the concentration profile and residual austenite profile relationship; in this case the function can be:

RA = 3.91C_C_+ 2.29 (2)

where RA is residual austenite in wt% and C_C_ is the carbon concentration in wt%.

R squared fit parameter calculated for Equation (2) was equal to 0.97. These approximations allow for further determination of the process parameters.

## 4. Discussion

The results obtained in the present work revealed a very good correlation between hardness (Figure 2), carbon content (Figure 3), and residual austenite content (Figure 4). Qualitatively, the plots shown follow the same trend, i.e., the highest values were obtained at the beginning of measurement, while increase in depth resulted in constant decrease of the measured values. Only a small variation between the hardness profile and carbon content depth profile was observed: at the beginning of hardness profile (approximately 100 µm) a slightly lower value of hardness was observed, about 730 HV; at a depth of 200 and 300 µm a hardness of 760 HV was observed. In parallel, no deviation in carbon content was observed in the region noted. The reason for this deviation between hardness profile and carbon profile follows. As observed in the image of microstructure of the material after carburizing, in the very outer part of the sample, formation of carbides is observed, while in the deeper part a uniform distribution of bright precipitates is observed (Figure 1). Thus, the main reason for the lower value of hardness is coarsening of precipitates. The reason why the GD-OES depth profile did not reveal lower carbon content (Figure 3) is that during GD-OES depth profiling, an area with a 4 mm diameter is sputtered, thus an average carbon content from the area noted is collected and measured. Therefore, for the GD-OES depth profiling, the size of precipitates does not play this detrimental role as in the case of hardness measurement. Considering this discussion, it can be concluded that the results of hardness and carbon concentration profile are in a very good agreement. It is clearly seen that the results of these two measurements are closely correlated. Thus, during mathematical modeling of the carburizing process, the concentration can be treated as interchangeable with the hardness of carbon in the steel. It would seem that the measured values of residual stress should follow the same trend. However, as shown in Figure 5, the residual stress distribution obeys a local minimum at a depth of 800 µm (Figure 5). This minimum cannot be correlated with lower carbon residual austenite content. Therefore, there should be another factor determining this phenomenon. As stated by Yang et al. [28], the martensite transformation temperature (Ms) decreases with the decrease of carbon content in austenite, so that they are lower in the carburized layer and higher in the core. This situation is shown schematically in Figure 6.

Thus it is obvious that the temperature during the quenching process is lower at the carburized layer and higher in the core. Moreover, the carbon content is higher in the carburized layer. The austenite–martensitic transformation starts at lower temperature T2 (point A, Figure 6) in the carburized layer and becomes higher (point B, Figure 6) in the core of the steel. The consequence of this situation is that in order to obtain martensitic transformation in a carburized layer, it must be cooled to a lower temperature than in the core of the steel. This situation leads to the formation of two fronts of martensitic expansion, which consequently causes the occurrence of a high compressive stress zone (more negative values of residual stresses). The measured residual stress distribution (Figure 5) reveals that this zone is located at a depth between 600 and 1000 µm. Combining the latter and the GD-OES depth profile (Figure 3), it is observed that a high compressive stress zone is present in the material containing between about 0.8 and 0.4 wt% of carbon.

## 5. Conclusions

In the present work, Pyrowear53 steel was subjected to the impulse carburizing LPC process. After carburization, the material was quenched and tempered. Postprocessing analyses included the measurement of hardness, carbon content, residual austenite, and residual stresses. The results indicate the following conclusions:

- Carburizing, quenching, and tempering processes increased the hardness of Pyrowear53 from 400 HV up to 750 HV in the carburized layer.
- The hardness profile obtained correlates with the carbon content and residual austenite profiles.
- The results of residual stress measurement revealed the presence of a local extremum at a depth of about 800 µm in the form of a zone showing the highest compressive stresses, despite no differences in hardness, carbon content, or residual austenite content.
- The occurrence of the extremum is explained by the formation of two fronts of martensite expansion, which result in the formation of a zone with higher compressive stresses at a depth between 600 and 1000 µm.

It was found that the presence of a zone with higher compressive stresses is observed in the material containing between 0.8 and 0.4 wt% carbon.

## Figures and Tables

**Table 1:** Nominal chemical composition of AMS 6308D and composition of Pyrowear53 steel measured using Spark-OES, wt%.

Element	Fe	C	Mn	Si	P	S	Cr	Ni	Mo	Cu	V
Nominal (wt%)	Bal. *	0.07–0.13	0.25–0.50	0.60–1.20	<0.015	<0.010	0.75–1.25	1.60–2.40	3.00–3.50	1.80–2.30	0.05–0.15
Measured (wt%)	Bal. *	0.13	0.41	0.93	0.011	0.003	1.04	1.97	3.23	1.92	0.1

* Bal. = Balance.

## Data Availability

The data are available from the corresponding author upon reasonable request.
